# Studies on Angiotensin-Converting Enzyme Insertion/Deletion Polymorphism and Genotype Distributions in Turkish Preeclampsia Patients

**DOI:** 10.1155/2012/108206

**Published:** 2012-03-25

**Authors:** Ceyhun Bereketoğlu, Mülkiye Kasap, Ayfer Pazarbaşı

**Affiliations:** Department of Medical Biology and Genetics, Faculty of Medicine, Cukurova University, 01330 Adana, Turkey

## Abstract

Placental, immune and genetic factors are thought to play an important role in preeclampia (PE)'s pathophysiology. Angiotensin-Converting Enzyme (ACE) plays a vital role in the renin-angiotensin-system (RAS) which regulates blood pressure by converting angiotensin I into a powerfull vasoconstrictor angiotensin II. A deletion polymorphism (D allele) has been reported to be associated with elevated ACE activity. The aim of the this study was to investigate whether there is an association between angiotensin converting enzyme (ACE) insertion/deletion (I/D) polymorphism and PE. In this study, 120 preeclamptic and 116 normotensive Turkish pregnant women were genotyped for ACE I/D polymorphism and the distribution of genotype and allele frequencies of this polymorphism in preeclampsia and controls were evaluated. Codominant, dominant and recessive models were appplied in ACE gene I/D polymorphism. In the codominant model, DD genotype was found significantly more frequent in preeclampsia than controls (*P* = 0.016). Moreover, in dominant model (DD frequency versus DI+II frequency) there was a significant relation between DD genotype and preeclampsia (*P* = 0.006). D allele frequency was 64.6% in preeclampsia while it was 56.1% in controls (*P* = 0.062). In conclusion, there was significant difference in genotype distribution between preeclampsia and controls.

## 1. Introduction

Preeclampsia (PE) is a disorder that occurs in women with a new-onset of hypertension and proteinuria after 20 weeks of pregnancy. It plays an important role in perinatal mortality and morbidity, as well as maternal mortality [1–8]. It affects 3–5% of all pregnancies worldwide, and the best treatment is delivery [4, 5, 9]. Although the aetiology of preeclampsia is still unclear, there are some evidences that preeclampsia is associated with abnormal placentation which is related to poor maternal defense mechanisms and impaired placentation in early gestation resulted from low-resistance uteroplacental circulation [10–12]. 

Angiotensin-converting enzyme (ACE, EC 3.4.15.1, a peptidyl carboxypeptidase) plays a vital role in the rennin angiotensin system (RAS) which regulates blood pressure by converting angiotensin I into a powerful vasoconstrictor angiotensin II. High ACE activity can contribute to hypertension because of its vasoconstriction effect [13, 14]. An insertion/deletion (I/D) polymorphism in the ACE gene occurs due to the insertion or deletion of an Alu 289 base pairs (bp) sequence located at intron 16 [15]. A deletion polymorphism (D allele) has been reported to be associated with elevated ACE activity [16]. Some investigators have reported in women from various geographical origins an association between the ACE D allele or DD genotype and increased risk of preeclampsia or pregnancy-induced hypertension [15–19], whereas others could not [20–23]. Women included in this study were all Caucasian.

The aim of the study was to investigate whether there is an association between ACE intron 16 I/D polymorphisms and PE.

## 2. Materials and Methods

Written approval was obtained from the Ethics Committee of Cukurova University Hospital and Baskent University, and all patients gave their informed consent before peripheral blood samples were taken. Information was enrolled retrospectively about one hundred twenty (120) preeclamptic women and one hundred fourteen (114) normotensive women with no history preeclampsia who delivered at two university hospitals located in Adana (Cukurova and Baskent University Hospitals) between September 2009 and August 2010. PE cases included both severe and mild PE, and all of them were early-onset cases (after 20 weeks). A total of 234 patients were studied. Clinical characteristics of the study population are reported in Table 1.

Preeclampsia was defined using the criteria of the National High Blood Pressure Education Program Working Group: (1) increase of 30 mm Hg or greater of systolic blood pressure, (2) increase of 15 mm Hg or greater of diastolic blood pressure (both criteria 1 and 2 refer to before and after 20 weeks of gestation), (3) if previous blood pressure was not known, a blood pressure must be ≥140 mm Hg for systolic and ≥110 mm Hg for diastolic after 20 weeks of gestation, (4) in addition to blood pressure, proteinuria was defined as the excretion of 0.3 g/L (1+ on a dipstick) or greater [24]. Women with a significant past medical history such as diabetes, chronic hypertension, pregnancies with malformed fetuses or infections, twin pregnancies were excluded from the preeclampsia patients and controls. The controls were normotensive women who had no history of preeclampsia and were recruited from the same centers randomly.

Maternal DNA was isolated from peripheral venous blood leukocytes using standard salting out method as previously described [25]. ACE intron 16 I/D polymorphism was genotyped by 2 PCRs using 3 primers [26]. The primers of first PCR were 5′-CTG GAG ACC ACT CCC ATC CTT TCT-3′ and 5′-GAT GTG GCC ATC ACA TTC GTC AGA T-3′. The 190 and 490 bp products of this PCR were from D and I alleles, respectively. The first amplification reaction was carried out in a total volume of 25 *μ*L, using 200 ng genomic DNA, 25 mM dNTPs, 10 pmol of each primer, 1 U Taq DNA polymerase (Vivantis), and 2.5 *μ*L ViBuffer S (Vivantis). PCR conditions were 94°C for 5 minutes, followed by 30 cycles at 94°C for 1 minute, 58°C for 1 minute, 72°C for 2 minutes, and a final step at 72°C for 4 minutes.

To avoid the misidentification of DI genotypes as DD, a second PCR was performed with the same antisense primer as in the first and different sense primer as 5-TTT GAG ACG GAG TCT CGC TC-3′ that generates a 408 bp fragment only in the presence of the I allele. The second PCR was carried out in a total volume of 25 *μ*L with the same reagents of the first amplification reaction. PCR conditions were 94°C for 5 minutes, followed by 40 cycles at 94°C for 1 minute, 60°C for 75 seconds, 72°C for 1 minutes, and a final step at 72°C for 10 minutes.

PCR products were analysed by 2% agarose gel after staining by ethidium bromide. Statistical analyses were carried out with the SPSS version 15.0. Pearson's chi-squared test was used for the statistical evaluation of the individual allele and genotype frequencies. The level of statistical significance was defined as *P* < 0.05. In the ACE I/D polymorphism, data were analyzed under three models: a codominant, a dominant, and a recessive model.

## 3. Results

The clinical characteristics of the study population are shown in Table 1. Mean maternal age was similar between controls and preeclampsia, while gestational age was significantly higher in controls than preeclampsia.

The genotype distributions and allele frequencies for ACE I/D polymorphism were summarized in Table 2. For ACE I/D polymorphism, the frequency DD genotype was 43.3% in preeclampsia, while it was 26.3% in controls analyzing data under a codominant model. In this model, the difference was found statistically significant (*P* = 0.016). Moreover, in the dominant model (DD frequency versus DI+II frequency), the difference between the two groups was found statistically significant (*P* = 0.006). The ACE D allele frequency was 64.6% in preeclampsia and 56.1% in controls, and the difference was not found statistically difference (*P* = 0.062).

## 4. Discussion

The present study showed an association between ACE DD genotype and preeclampsia in Turkish population. In the analyzed Turkish population, PE cases included both severe and mild PE and all of them were early-onset cases (after 20 weeks). On average, deliveries occurred about 3 weeks earlier in the preeclamptic women than in the controls.

The results of previous studies on association of ACE I/D polymorphism with preeclampsia were conflicting presumably attributable to differences in study population, genetic backgrounds, and size of study groups. Some studies showed significantly higher incidence of DD genotype and/or D allele in preeclampsia and/or pregnancy-induced hypertension [15–19]. On the other hand, no difference in genotype distribution and allele frequency and no association between DD genotype and occurrence of preeclampsia were found in some studies [20–23]. A possible reason for the inconsistency among these reports may be a genetic basis that causes different susceptibilities among different populations.

In the present study, for ACE I/D polymorphism analysis, we used three models: codominant, recessive, and dominant models which were used in a study on Caucasian population [15]. We detected differences in genotype distribution between preeclampsia and controls for the ACE gene polymorphism when data were evaluated using a dominant model, considering DD frequency versus DI+II and using a codominant model. In both models, we found a higher incidence of DD genotype in preeclampsia when compared to controls. However, in the previous study on caucasian population, a higher difference was found when data were evaluated using a recessive model, considering II frequency versus DI+DD frequency [15]. Moreover, we found no association between allelic frequency and preeclampsia. A weakness of our study is that the data observed was obtained from a limited population (Turkish women). The main limitation of the study is the lack of the circulating cytokine measures that are needed to prove a functional relationship between polymorphisms, elevated cytokines, and PE.

In conclusion, although our results show an association between the ACE DD genotype and preeclampsia in the analyzed Turkish population, further studies using a larger number of subjects and analyses that include genetic, environmental, and other potential factors are needed to confirm these results.

## Figures and Tables

**Table 1:** The clinical characteristics of the study population and controls.

	Mean of maternal age (years)	Mean of maternal weight (kg)	Number of pregnancy	Gestational age (weeks)	Systolic blood pressure (mm Hg)	Diastolic blood pressure (mm Hg)
PE	29,00 ± 7,044	78,89 ± 10,201	2,29 ± 1,597	34,615 ± 4,698	151,56 ± 16,116	98,85 ± 12,934
Controls	27,41 ± 5,317	77,01 ± 9,985	2,27 ± 1,639	37,060 ± 4,095	110,54 ± 10,000	71,63 ± 8,044

**Table 2:** Angiotensin-converting enzyme (ACE) polymorphism in normal pregnancies and in pregnancies complicated by preeclampsia (PE).

Model	Controls	PE	*P* value
	*n* = 114	*n* = 120	
	Distribution	Distribution	
Codominant			

DD	30 (26,3%)	52 (43,3%)	0.016
DI	68 (59,6%)	51 (42,5%)	
II	16 (14,0%)	17 (14,2%)	

Recessive			

II	16 (14,0%)	17 (14,2%)	0.977
DD+DI	98 (86,0%)	103 (85,8%)	

Dominant			

DI+II	84 (73,7%)	68 (56,7%)	0.006
DD	30 (26,3%)	52 (43,3%)	

Allele			

D	128 (56,1%)	155 (64,6%)	0.062
I	100 (43,9%)	85 (35,4%)	
